# Circulating Undercarboxylated Osteocalcin as Estimator of Cardiovascular and Type 2 Diabetes Risk in Metabolic Syndrome Patients

**DOI:** 10.1038/s41598-020-58760-7

**Published:** 2020-02-04

**Authors:** Blanca Riquelme-Gallego, Laura García-Molina, Naomi Cano-Ibáñez, Guillermo Sánchez-Delgado, Francisco Andújar-Vera, Cristina García-Fontana, Sheila González-Salvatierra, Enrique García-Recio, Virginia Martínez-Ruiz, Aurora Bueno-Cavanillas, Manuel Muñoz-Torres, Beatriz García-Fontana

**Affiliations:** 1 Department of Preventive Medicine and Public Health, University of Granada, Granada, Spain; 2 Fundación para la Investigación Biosanitaria de Andalucía Oriental (FIBAO), Granada, Spain; 3 Instituto de Investigación Biosanitaria de Granada (ibs.GRANADA), Granada, Spain; 4 CIBER of Epidemiology and Public Health (CIBERESP), Carlos III Institute of Health, Madrid, Spain; 5 PROFITH “PROmotingFITness and Health through physical activity” Research Group, Department of Physical Education and Sport, Faculty of Sport Sciences, University of Granada, Granada, Spain; 6 Department of Medicine, Faculty of Medicine, University of Granada, Granada, Spain; 7 Nursing Department, Faculty of Health Sciences, University of Granada, Granada, Spain; 8 Bone Metabolic Unit, Endocrinology and Nutrition Division, San Cecilio University Hospital, Granada, Spain; 9 CIBER of Fragility and Healthy Aging (CIBERFES), Carlos III Institute of Health, Madrid, Spain

**Keywords:** Biomarkers, Endocrinology, Risk factors

## Abstract

Undercarboxylated osteocalcin (ucOC) could be a biomarker of glucose disturbances and cardiovascular risk. Our study aimed to determine the association between serum levels of ucOC and cardiovascular risk in metabolic syndrome (MetS) patients and to analyse its potential role as estimator of type 2 diabetes (T2D) risk in this population. This cross-sectional study included 235 patients with MetS, 53.2% women, aged 55–75 years. Circulating ucOC levels were measured by ELISA. Cardiovascular risk was determined as Z-score of the diagnostic criteria for MetS (CV-ZS). Linear regression model was performed to analyse the association between circulating ucOC and CV-ZS. A receiver operating curve (ROC) was performed to analyse the usefulness of ucOC as T2D risk estimator. Patients above the CV-ZS median showed significant lower ucOC levels. We found an inverse association between ucOC levels and CV-ZS in MetS patients without T2D. Patients with ucOC levels below the 25^th^ percentile showed worse cardiometabolic profile and higher cardiovascular and T2D risk. The area under the curve performed better when ucOC levels were included along with the classic T2D risk factors. The measurement of circulating ucOC could be a useful tool to identify increased cardiovascular and T2D risk in MetS patients without T2D.

## Introduction

Current lifestyle is causing a remarkable increase in overweight up to epidemic numbers globally^1^. In Spain, over 60% of the adult population is overweight or obese^2^. According to this trend, an increase of 16% in the number of cases is estimated by 2030 associated with a 58% increase in direct healthcare costs^3^. A large body of evidence has shown that all-cause mortality, and especially cardiovascular-related mortality, is associated with an increased central adiposity and overweight^4^. Visceral obesity in conjunction with other disorders, such as dyslipidaemia, hypertension and fasting hyperglycaemia lead to the metabolic syndrome (MetS), conferring thus a larger risk of developing cardiovascular disease (CVD)^5^.

An association between osteoporosis, CVD and cardiovascular-related mortality has been reported^6,7^. The common risk factors involved in bone fragility and CVD could partially explain this association. Therefore, the imbalance between bone formation and resorption occurring in bone disorders could play a role in the development of vascular complications^8^. Recent data suggest an influence of bone metabolism on energy balance, which may be relevant for CVD^9^. In this regard, angiogenesis plays a major role in bone fracture healing and repair^10^ and changes in the local vasculature are associated with the progression of numerous conditions affecting bone, such as osteoporosis, rheumatoid arthritis, bone cancer and metastasis^11^.

Bone-related proteins, such as osteocalcin (OC), are of special interest for the study of CVD. OC is a small non-collagenous protein of 49 amino acids produced exclusively by osteoblasts and it is one of the most abundant proteins in bone. OC has been classically linked to bone mineralisation^12^. Moreover, the role of OC as an endocrine hormone involved in the regulation of energy metabolism has been demonstrated *in vitro* and in animal models^13^. Most OC is incorporated into the extracellular bone matrix; however, its undercarboxylated fraction (ucOC) is released into the bloodstream. UcOC can act directly on pancreatic beta cells and on adipocytes, regulating insulin secretion and insulin sensitivity. These findings have assigned a new role to the bone as an endocrine organ with extra-skeletal functions^14^.

Several studies in humans have evaluated the relationship between circulating levels of total OC and impaired glucose metabolism^15^, pointing to OC as a marker that could predict changes in glucose homeostasis^16^.

Moreover, OC has been linked to atherosclerotic parameters, such as brachial-ankle pulse wave velocity and intima-media thickness in patients with T2D^17^ and previous atherosclerotic disease^8^.

Despite the large evidence about OC as a metabolic regulator^18–20^, the involvement of ucOC in cardiovascular risk in MetS patients remains unclear. This occurs because most of the studies in this regard have analysed the relationship between ucOC and individual cardiovascular parameters. To date, no studies evaluating the role of ucOC serum levels as a potential biomarker of cardiovascular risk determined as a global score are available.

In this context, the aims of the present study were: 1. To determine the serum levels of ucOC in adults with MetS in order to analyse the association between ucOC and cardiovascular risk scores. 2. To provide information on the possible usefulness of circulating ucOC level as estimator of T2D risk in this population.

## Results

### Characteristics of the study population

The clinical characteristics of the entire population according to the cardiovascular risk Z-score (CV-ZS) for 50^th^ percentile (P50) are summarised in Table 1.

**Table 1:** Anthropometric and biochemical parameters of the study population in the total sample according to P50 of CV-ZS.

	Total sample (N = 235)		<P50 CV-ZS (N = 117)		>P50 CV-ZS (N = 118)		*p-*value
Men/Women	111/124		52/65		65/59		0.394
Sedentarism (%)	44.7%		41.0%		48.3%		0.262
Current smoker (%)	11.1%		8.5%		13.6%		0.221
Presence of T2D (%)	22.6		11.2%		36.1%		**<0.001**
Antidiabetic drugs (%)	23		12.0%		33.9%		**<0.001**
Antihypertensive drugs (%)	75.3		70.1%		80.5%		0.064
Hypolipidaemic drugs (%)	37.4		41.9%		33.9%		0.207
	**mean**	**SD**	**mean**	**SD**	**mean**	**SD**	
Age (years)	63.9	4.9	64.4	4.8	63.6	4.9	0.211
BMI (m/kg^2^)	32.5	3.5	30.9	2.9	34.0	3.4	**<0.001**
WC (cm)	107.7	10.2	102.9	8.4	112.4	9.6	**<0.001**
Mean BP (mm Hg)	103.3	11.4	99.4	9.8	107.2	11.6	**<0.001**
FPG (mg/dL)	102.3	25.3	91.5	12.2	113.0	30.0	**<0.001**
Total cholesterol (mg/dL)	202.3	36.9	206.8	36.1	197.8	37.2	0.062
HDL cholesterol (mg/dL)	49.1	10.0	53.4	9.8	44.8	8.3	**<0.001**
LDL cholesterol (mg/dL)	124.3	33.9	126.6	31.5	122.1	36.1	0.309
TG levels (mg/dL)	169.7	74.1	141.9	49.9	197.3	83.4	**<0.001**
HbA1c (%)	6.0	1.1	5.6	1.1	6.3	0.9	**<0.001**
ucOC (ng/mL)	5.9	5.1	6.9	6.0	4.9	3.7	**0.004**
Log ucOC (ng/mL)	0.6	0.3	0.7	0.3	0.6	0.3	**<0.001**
Framingham (%)	14.38	7.37	12.55	7.11	16.26	7.20	**<0.001**
REGICOR (%)	6.27	3.15	5.21	2.30	7.31	3.51	<0.001

BMI: body mass index; WC: waist circumference; FPG: fasting plasma glucose; TG: triglyceride; T2D: type 2 diabetes; ucOC: undercarboxylated osteocalcin; CV-ZS: cardiovascular risk score.

Data for continuous variables are expressed as mean ± SD. Data for categorical variables are expressed as percentages. Comparison was performed between groups with CV-ZS below and above P50. Student’s t test and χ^2^ test were used for comparisons of continuous and categorical variables, respectively, between groups. Significance was set at *p* < 0.05.

Both groups (<P50 CV-ZS and >P50 CV-ZS) were comparable in age and sex. As expected, the MetS patients with higher cardiovascular risk (>P50 CV-ZS) showed significantly worse cardiometabolic profile in terms of body mass index (BMI), waist circumference (WC), blood pressure (BP), fasting plasma glucose (FPG), high-density lipoprotein cholesterol (HDL-C), triglyceride (TG) levels and glycated haemoglobin (HbA1c). In addition, these patients showed significantly lower serum levels of ucOC than the group with CV-ZS below P50.

**Figure 1:**
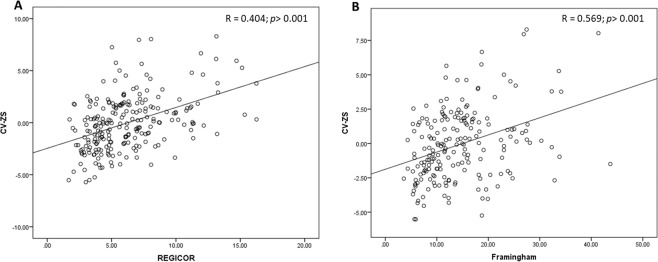
Scatter plots showing the correlation (Pearson’s test) between CV-ZS and REGICOR score (**A**) and between CV-ZS and Framingham score (**B**) in MetS patients.

Considering cardiovascular risk scores estimated by Framingham and REGICOR, we found significant differences between groups according to P50 of CV-ZS, finding a significant positive correlation between CV-ZS and Framingham and REGICOR scores (Fig. 1).

We found significantly higher percentage of patients with prevalent T2D in the group above P50 of CV-ZS than in the group below P50 of CV-ZS.

### Association between serum levels of ucOC and cardiovascular risk factors

Our results showed a correlation between the logarithm of serum ucOC levels and sex (r = 0.191, *p* = 0.004). Women showed higher serum levels of ucOC than men (0.70 ± 0.33 ng/mL *vs*. 0.57 ± 0.33 ng/mL, *p* = 0.004). No association was found between ucOC levels and age (r = 0.041, *p* = 0.535).

**Figure 2:**
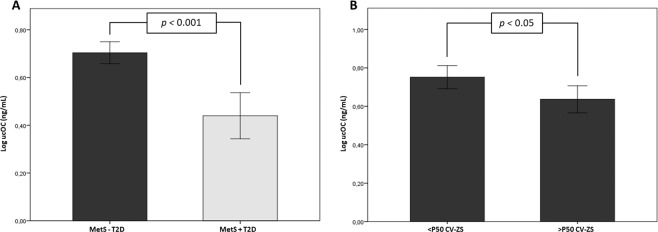
Bar graphs representing serum levels of ucOC in MetS patients with type 2 diabetes (+T2D) or without T2D (-T2D) (**A**) and in MetS patients without T2D based on the median distribution of CV-ZS (**B**). Data are represented as mean ± 95% confidence interval (CI). Student’s t test was used for comparisons between groups. Significance was set at *p* < 0.05.

When patients were further divided according to the presence of T2D, we found that serum ucOC levels were significantly lower in T2D patients than in MetS patients without T2D (Fig. 2A). To prevent possible biases in the results related to the potential influence of T2D on serum levels of ucOC, the group of patients with MetS without T2D was analysed separately. In these patients, those presenting a higher cardiovascular risk (>P50 CV-ZS) showed significantly lower levels of ucOC than the group with lower cardiovascular risk (Fig. 2B). This trend remained close to significance after sex adjustment (0.65 ± 0.03 ng/mL *vs*. 0.75 ± 0.03 ng/mL; *p* = 0.074).

The association between circulating ucOC levels and cardiovascular risk factors showed a positive correlation between circulating levels of ucOC, HDL-C and total cholesterol and an inverse correlation between ucOC levels and WC, FPG, HbA1c and cardiovascular risk scores (CV-ZS, Framingham and REGICOR) in the entire sample. When T2D patients were excluded from the analysis, no association was found between ucOC and the variables related to glucose homeostasis (FPG and HbA1c). Nevertheless, the association between ucOC and WC, HDL-C, and cardiovascular risk scores remained significant (Table 2).

**Table 2:** Pearson correlation coefficients between serum levels cardiovascular risk factors and cardiovascular risk scores in the entire sample and in MetS patients without type 2 diabetes (-T2D).

	Log ucOC Total sample		Log ucOC MetS -T2D	
	r	*p*	r	*p*
WC	−0.163	0.014	−0.169	0.026
HDL cholesterol	0.281	<0.001	0.295	<0.001
FPG	−0.186	0.005	−0.060	0.426
Total cholesterol	0.143	0.032	0.092	0.226
HbA1c	−0.153	0.040	0.035	0.676
CV-ZS	−0.225	0.001	−0.189	0.012
Framingham	−0.308	<0.001	−0.191	0.022
REGICOR	−0.192	0.004	−0.199	0.008

ucOC: undercarboxylated osteocalcin; WC: waist circumference; FPG: fasting plasma glucose; CV-ZS: cardiovascular risk score. Significance was set at *p* < 0.05.

### Relationship between serum levels of ucOC and cardiovascular risk

To determine the independent effect of circulating ucOC levels on cardiovascular risk expressed as CV-ZS (dependent variable), a multiple backward model of linear regression analysis was performed adjusting for the effect of cardiovascular risk-associated variables (age, sex, smoking status and sedentarism) and medication (hypolipidaemic, antidiabetic and antihypertensive drugs). These results showed that the only variables associated with CV-ZS were ucOC levels (B = −1.317, [−2.417/−0.217], *p* = 0.019) and hypolipidaemic drugs (B = −0.891, [−1.613/−0.169], *p* = 0.016), regardless of the effect of the other variables in MetS patients without T2D.

### Evaluation of cardiovascular risk factors according to the 25^th^ and 50^th^ percentiles of ucOC serum levels

In order to establish the serum level of ucOC that determines significant differences in the metabolic profile and cardiovascular risk of the study population, MetS patients with and without T2D were divided into subgroups based on the percentiles of the ucOC levels. A comparative sex-adjustment study was performed according to the 25^th^ and the 50^th^ percentiles. Although statistically significant differences were found in some risk factors when study groups were divided according to the 50^th^ percentile, the largest differences were found when the 25^th^ percentile was considered in the whole sample. Therefore, MetS patients below 25^th^ percentile of ucOC serum levels showed a significantly worse cardiometabolic profile and higher cardiovascular risk in terms of cardiovascular risk scores (CV-ZS, Framingham and REGICOR).

This trend of worse cardiometabolic profile associated with lower levels of ucOC was maintained in MetS patients without prevalent T2D. However, significant differences were found only between ucOC and HDL-C. In a similar way that with the entire population, patients below the 25^th^ percentile of ucOC serum levels had significantly higher cardiovascular risk in terms of CV-ZS and REGICOR scores (Table 3).

**Table 3:** Comparison of cardiovascular risk parameters according to the 25^th^ and 50^th^ percentiles (P25 and P50, respectively) of serum ucOC logarithm by sex-adjusted univariate analysis of variance.

Total sample	P25 of ucOC levels					P50 of ucOC levels				
	<2.53 ng/mL (n = 57)		≥2.53 ng/mL (n = 170)		*p*	<4.58 ng/mL (n = 114)		≥4.58 ng/mL (n = 113)		*p*
	mean	SD	mean	SD		mean	SD	mean	SD	
BMI	32.73	3.37	32.41	3.61	0.535	32.58	3.23	32.41	3.85	0.701
WC	109.78	9.80	106.88	10.27	0.260	108.77	10.01	106.44	10.31	0.353
mean BP	103.74	12.16	103.17	11.15	0.819	103.25	11.26	103.38	11.56	0.488
HDL cholesterol	45.35	7.79	50.76	10.28	**0.002**	47.31	9.13	51.52	10.39	**0.009**
FPG	111.63	34.29	99.05	20.86	**0.001**	106.11	28.16	98.27	21.76	**0.013**
TG	192.19	98.63	162.47	63.88	**0.008**	178.17	82.87	161.63	65.51	**0.090**
HbA1c	6.33	0.99	5.84	1.08	**0.006**	6.09	0.94	5.86	1.19	0.126
CV-ZS	1.06	3.38	−0.40	2.41	**0.000**	0.42	2.95	−0.49	2.47	**0.011**
Framingham	17.56	8.29	13.19	6.75	**0.001**	15.66	8.01	12.84	6.41	**0.015**
REGICOR	7.11	4.01	5.95	2.74	**0.055**	6.67	3.55	5.80	2.61	0.133
**MetS – T2D group**	**P25 of ucOC levels**					**P50 of ucOC levels**				
	**<3.00 ng/mL (n = 44)**		**≥3.00 ng/mL (n = 131)**		***p***	**4.91 ng/mL (n = 88)**		**≤4.91 ng/mL (n = 87)**		***p***
	**mean**	**SD**	**mean**	**SD**		**mean**	**SD**	**mean**	**SD**	
BMI	32.75	3.16	32.37	3.73	0.743	32.57	3.32	32.40	3.82	0.697
WC	109.79	9.81	106.51	10.26	0.277	108.50	9.75	106.49	10.70	0.959
mean BP	104.64	11.53	102.64	11.29	0.421	103.46	11.49	103.12	11.32	0.789
HDL cholesterol	45.92	7.86	51.16	10.49	**0.006**	47.73	9.24	51.53	10.52	**0.038**
FPG	109.54	31.18	98.52	21.14	0.509	105.29	27.02	98.29	22.79	0.167
TG	184.22	93.15	162.74	63.14	0.196	176.57	80.90	161.50	66.27	0.162
HbA1c	6.19	0.97	5.86	1.12	0.438	6.04	0.90	5.89	1.26	0.373
CV-ZS	0.96	3.15	−0.53	2.39	**0.010**	0.31	2.91	−0.47	2.49	**0.097**
Framingham	16.57	8.15	13.17	6.75	0.726	15.50	7.82	12.71	6.50	0.481
REGICOR	7.03	3.82	5.85	2.67	**0.029**	6.54	3.44	5.86	2.68	0.353

BMI: body mass index; WC: waist circumference; FPG: fasting plasma glucose; TG: triglyceride; ucOC: undercarboxylated osteocalcin; CV-ZS: cardiovascular risk score. Data for continuous variables are expressed as mean ± standard deviation (SD). Univariate analysis of variance using a general linear factorial model adjusted by sex was used for comparisons between quantitative variables. Significance was set at *p* < 0.05.

### Usefulness of ucOC serum levels to estimate the T2D risk in MetS patients

A logistic regression model was performed to assess the variables related to T2D risk in MetS patients. The independent variables included in the multiple logistic regression model were those anthropometric and biochemical variables biologically linked to T2D development (age, sex, sedentarism, HDL-C and LDL cholesterol, TG levels, FPG and WC), in addition to ucOC levels categorised by the 25^th^ percentile cut-off point of the total sample (2.53 ng/mL). We found that the serum levels of ucOC were an independent estimator of T2D risk in patients with MetS (OR = 6.39, [2.51/16.27], *p* < 0.001) in addition to FPG levels (OR = 1.08, [1.05/1.10], *p* < 0.001).

**Figure 3:**
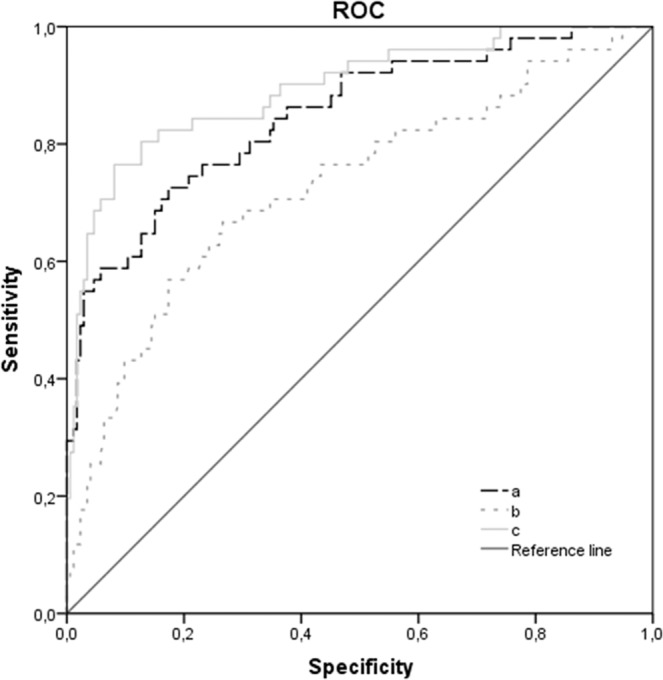
ROC curve for the usefulness of ucOC level as an estimator of T2D in MetS patients. (**a**) age, sex, WC, FPG, TG levels, HDL and LDL cholesterol; AUC = 0.848; *p* < 0.001. (**b**) logarithm of ucOC serum levels; AUC = 0.727; *p* < 0.001. (**c**) age, sex, WC, FPG, TG levels, HDL and LDL cholesterol + log ucOC serum levels; AUC = 0.894; *p* < 0.001. T2D: type 2 diabetes; WC: waist circumference; FPG: fasting plasma glucose; TG: triglyceride; ROC: receiver operating curve; AUC: area under the curve.

A receiver operating curve (ROC) analysis was performed to assess the usefulness of serum levels of ucOC as a marker of T2D risk. Three different models were assessed. The first model included the main T2D risk factors (age, sex, WC, FPG, TG levels, HDL-C and LDL cholesterol) (Area under the curve (AUC) = 0.848; *p* < 0.001). The second model included only the logarithm of serum levels of ucOC (AUC = 0.727; *p* < 0.001). Finally, the model that combined serum levels of ucOC and T2D risk factors showed the highest AUC value (AUC = 0.894; *p* < 0.001) (Fig. 3).

## Discussion

Our results show that ucOC serum levels are independently associated with cardiovascular risk determined as Z-score in MetS patients without prevalent T2D. We found that lower circulating levels of ucOC are related to worse metabolic profile and higher cardiovascular risk in MetS patients regardless of the presence of T2D. Furthermore, we found a relationship between serum levels of ucOC, sex, FPG and lipid profile in the whole population and with WC and HDL-C cholesterol in patients with MetS without T2D. Finally, serum levels of ucOC may act as an independent estimator of T2D risk in MetS patients.

The involvement of total OC and ucOC levels on energy metabolism and their close association with glucose and lipid metabolism has been largely studied^21^. Several studies described the inverse association between circulating OC and the presence of T2D^16,22,23^, insulin resistance^24,25^, and measures of adiposity^26^. Most of the studies in MetS patients outlined the decrease in total OC levels in MetS patients compared to healthy subjects^18,27–29^. Nevertheless, other authors found no differences in total OC levels in postmenopausal women diagnosed with MetS compared to those without MetS^23^. Regarding ucOC levels, few data are available in patients with MetS.

Our findings showed lower ucOC serum levels in MetS patients at higher cardiovascular risk, regardless of the presence of T2D. Among patients with MetS, those with prevalent T2D showed worse cardiometabolic profile and lower serum levels of ucOC. This finding is supported by validated cardiovascular risk scores, such as Framingham and REGICOR scores for the Spanish population, showing that patients above 50^th^ percentile of cardiovascular risk scores had lower ucOC levels and more unfavourable biochemical and anthropometric profiles. In consistency with our results, recent studies have reported lower serum levels of ucOC in T2D patients than in healthy subjects^30^ and lower in T2D patients with MetS than in those without MetS^31^.

MetS diagnosis implies an increase in cardiovascular risk^32^. However, a high variability of cardiovascular risk is found among these patients. Thus, within the MetS population, some patients are metabolically “healthier” than those with more unfavourable analytical and anthropometric values who would have worse prognosis. This fact makes difficult to categorise the cardiovascular risk in MetS patients. Despite tools to estimate cardiovascular risk, such as Framingham or REGICOR scores, are available, both of them present some limitations, such as the non-inclusion of important risk parameters, such as WC, the variability of parameters included depending on the calculator used, the loss of cases due to the lack of qualifying options, or the subjectivity of patients, among others. Therefore, these tools have been used primarily for epidemiological studies. In this way, it would be very useful to have a tool based on the diagnostic criteria of MetS reflecting the “metabolic” cardiovascular risk regardless of the risk due to environmental factors, such as lifestyle and the current medication (“acquired” cardiovascular risk).

In this regard, we calculated a CV-ZS to homogenise and to stratify the cardiovascular risk in this population considering the risk factors for MetS diagnostic accepted by the US National Cholesterol Education Program Adult Treatment Panel III (NCEP ATP III)^33^. This score shows a close correlation with validated cardiovascular risk scores, such as Framingham and REGICOR.

The relationship between total OC levels and cardiovascular parameters has been previously explored showing an association between lower serum levels of OC and atherosclerotic parameters in patients with T2D^8,34^ and myocardial infarction in young patients^35^, as well as a lower mortality rate associated with increased total OC levels^36^.

In the MetS population, some studies associate lower levels of total OC with cardiovascular risk parameters^30,37,38^ and with abdominal aortic calcification in men^39^.

Although some studies have linked ucOC levels to the risk of MetS through its relationship with individual cardiovascular risk factors^18,40–42^; to date, no studies evaluating the role of circulating ucOC as estimator of generalised cardiovascular risk in MetS patients are available. Our results show that ucOC serum levels could be an important estimator affecting cardiovascular risk expressed as CV-ZS, after adjusting by lifestyle and medication-related variables, only in those MetS patients who do not meet T2D criteria. This result could be explained because the presence of diabetes is one of the major determinants of cardiovascular risk rather than OC levels. In consistency with our results, some studies have shown that a higher ratio of circulating ucOC/OC was independently associated with lower incidence of myocardial infarction in older men^43^. Contrary to our results, a cross-sectional study including 162 Korean subjects showed a relationship between higher serum levels of ucOC and coronary artery calcification^44^. These differences could be due to the fact that their study population included also diabetic patients, but no adjustment for the presence of diabetes or any type of medication was performed.

The association between ucOC levels and cardiovascular risk could be explained, in part, by its relationship with some cardiovascular factors, such as WC, HDL-C, total cholesterol, FPG and HbA1c. In MetS patients without T2D, HDL-C was the main variable associated with serum ucOC levels. These relationships have been previously reported in other studies^16,30,31^. One of these studies suggested that higher levels of HDL-C could influence a higher OC production^16^. According to this, higher levels of ucOC found in MetS patients with lower CV-ZS could be related to higher levels of HDL-C.

Our results showed an association between serum levels of ucOC and sex, being these levels higher in women than men, in consistency with the previously reported^45^. However, the cause of this sex-based difference has not been explored yet. We suggest that higher ucOC levels found in women could be explained by the increase in bone resorption leading to bone loss during menopause. Bone degradation could increase serum levels of ucOC. An alternative hypothesis that we suggest states that increased levels of ucOC may act as a compensatory mechanism to restore bone homeostasis in postmenopausal women.

In contrast with the studies reporting an association between OC and ucOC levels and age^46,47^, we found no association in our study population, neither for men nor for women. Accordingly, De Pergola *et al*., also found no association between serum levels of OC and age neither in obese nor in T2D patients^16^. The lack of association between ucOC levels and age could be due to the restricted inclusion criteria on age, since our study population aged 55–75 years.

Based on the inverse and independent association between serum ucOC levels and CV-ZS found in our study, we determined that MetS patients with serum ucOC values below 2.53 ng/mL would have a worse metabolic profile than those with levels above this cut-off point. This trend was maintained for the group of MetS patients without prevalent T2D, but significant differences were found only for HDL-C levels. As expected, all patients below this cut-off point had significantly higher cardiovascular risk in terms of CV-ZS and REGICOR related to lower ucOC levels, regardless of diabetes.

We suggest that the measurement of ucOC serum levels could be useful for the estimation of cardiovascular risk in MetS patients. Accordingly, Alfadda A. *et al*.^31^ suggested that ucOC levels could play a role in the evaluation of the cardiovascular risk in patients with T2D due to the multiple associations found between ucOC levels and individual cardiovascular risk factors. However, this is the first study that determines an unified Z-score including the main cardiovascular risk factors to classify MetS patients according to their “metabolic” cardiovascular risk.

Additionally, MetS patients have a larger risk of developing T2D associated with their metabolic profile. The association between ucOC levels and glucose homeostasis is consistent and there is a large evidence reporting the role of ucOC as a regulator of glucose and energy metabolism^48,49^. Higher levels of this protein have been related to an improvement in insulin sensitivity and secretion due to the direct action of ucOC on adipocytes and β-cell mass^50^. As above mentioned, within a population of MetS patients, there are patients at higher risk than others to become diabetic patients depending on their metabolic profile. Considering ucOC levels as an independent variable in conjunction with biological variables related to T2D development, we found that the risk of T2D development in MetS patients with ucOC serum levels below 2.53 ng/mL was 6-fold higher than in those with serum levels of ucOC above this value. Our ROC curve analysis revealed that the inclusion of ucOC serum levels in addition to age and T2D-related variables, improves the prediction model for T2D risk in MetS patients. Similarly, to our results, Villafan-Bernal *et al*. used the ucOC/OC index to predict the probability of having T2D in a cohort of T2D patients and healthy subjects. Patients with ucOC/OC index below 0.31 had 12.6-fold increased probability of developing T2D than patients with higher index^51^. These findings strengthen our above-mentioned results, suggesting that serum levels of ucOC could be a potential biomarker to estimate cardiovascular and T2D risk in MetS patients. In addition, based on the aforementioned evidences, in the future, ucOC might become a therapeutic target to prevent cardiovascular events as reported by recent studies conducted in animal models^52,53^.

Our study has certain limitations. First, the cross-sectional design precludes any determination of causality in our findings. Second, we did not measure total OC, N-MID OC or other factors that could affect serum levels of ucOC, such as vitamins D and K, which would add valuable information to our results. Third, the collection of sedentary lifestyle data was recorded using a self-administered questionnaire, which implies the possibility of bias. Finally, since our study was conducted in a specific population of MetS patients, we cannot ascertain that the same results would be found in other ethnic or study groups.

The strengths of this study lie on the novel evaluation of cardiovascular risk in MetS patients using a global and unified score of accepted MetS risk factors, allowing thus a better characterisation of the cardiovascular risk in this heterogeneous population. In addition, we have considered potential confounders, such as the presence of T2D and the most commonly used medication in MetS patients.

To our knowledge, this is the first study showing a robust relationship between serum levels of ucOC and cardiovascular risk score. Moreover, the large sample size provides sufficient statistical power giving robustness to our results.

In summary, we suggest that circulating ucOC levels could be an estimator of the cardiovascular risk in MetS patients without T2D. In addition, lower serum levels of ucOC could contribute to T2D development. The measurement of circulating ucOC levels could become a strategy to identify MetS patients at high risk in order to establish preventive and therapeutic approaches. These findings break new ground for new research lines to evaluate the role of ucOC as a biomarker and as a potential therapeutic target, as well as the usefulness of CV-ZS in clinical practice in future longitudinal studies.

## Methods

### Study population

A cross-sectional study was conducted in 235 patients with MetS, aged 55–75 years (53.2% women). All patients included were overweight or obese (BMI ≥ 27 kg/m²) and met at least three diagnostic criteria for MetS: high BP, impaired FPG level, high TG level, low HDL-C level and increased WC according to the definitions of the NCEP ATP III^33^. A total of 21.8% of the study patients had prevalent T2D, diagnosed according to the American Diabetes Association criteria.

From December 2014 to December 2016, patients were consecutively recruited at primary healthcare centres in Granada (Spain). All patients were Caucasian outpatients, and they did not present any prevalent cardiovascular disease, cancer, morbid obesity (≥40 kg/m²), and/or presence of bone diseases that could interfere with the study protocol. None of them had been treated with calcium or vitamin D supplements, hormone therapy, anti-osteoporotic drugs, steroids, vitamin K antagonists, thiazolidinediones, glucocorticoids or other medications that might affect bone metabolism.

The study was conducted with the approval of the ethics committee of the San Cecilio University Hospital of Granada and conformed to the principles of the World Medical Association’s Declaration of Helsinki. Written informed consent was obtained from all patients.

### Clinical and anthropometric evaluation

Anthropometric data were collected according to standard procedures. The BMI was calculated using the Quetelet formula (weight in kilograms divided by the square of height in meters); WC was measured midway between the superior border of the iliac crest and the lowest rib. Systolic BP (SBP) and diastolic BP (DBP) were obtained using a standard mercury sphygmomanometer (12 cm long and 35 cm wide). The mean BP was calculated using the equation (2 x DBP + SBP)/3)^54^.

Patients reported smoking status and level of physical activity by using a specific health questionnaire. Smoking status was categorised as non-smoker or current smoker. Physical activity was recorded using a specific questionnaire in which study patients reported how many hours used to spend on each activity throughout the day. Based on the results, the study sample was divided into two groups: sedentary (more than 7 hours sitting per day) and non-sedentary (less than 7 hours sitting per day).

### Biochemical measurements

Blood samples were collected after an overnight fast. Conventional analyses of lipid profile (total cholesterol, HDL-C, LDL-cholesterol and TG level), FPG and glycated haemoglobin (HbA1c) were determined at the Clinical Analysis Unit of San Cecilio University Hospital of Granada. The levels of ucOC were measured by enzyme-linked immunosorbent assay (ELISA) according to the manufacturer’s instructions (Takara Bio, Japan). All measurements were determined in duplicate at our laboratory. Precision testing was performed by the determination of intra-assay and inter-assay variations (6% and 10%, respectively), which were consistent with those reported by the manufacturer (5.21% and 8.33%, respectively).

### Calculation of cardiovascular risk scores

A Z-score of the combined cardiovascular risk factors used for the diagnosis of MetS (CV-ZS) was calculated including WC, mean BP, HDL-C, TG levels, and FPG according to the NCEP ATP III. The Z-score of each cardiovascular risk factor was calculated using the mean and standard deviation (SD) by applying the equation (x – mean (x))/SD), being x the variable of interest. The mean-centring and SD normalisation were sex-specific for each variable. The CV-ZS was the average of the Z-scores of TG levels, FPG, WC, mean BP and the inverse Z-score of HDL-C^55,56^.

In order to correlate the CV-ZS with other validated cardiovascular risk scores, the Framingham and REGICOR (for Spanish population) scores were estimated according to the equation described by Wilson P.W. *et al*.^57^ and through the online calculator available at www.imim.cat/ofertadeserveis/software-public/regicor/?1^58^, respectively. Both scores include age, sex-adjusted cardiovascular risk variables (total cholesterol, HDL-C and SBP and DBP), and presence of diabetes and tobacco consumption (both variables collected using a questionnaire).

### Statistical analysis

Analyses were performed using SPSS version 22.0 software (SPSS, Inc., Chicago, IL). Continuous variables were expressed as mean ± SD, and categorical variables were expressed as percentages. Kolmogorov-Smirnov test was used to test the normality of the variables. A log transformation was performed for skewed variables. Comparisons of continuous variables among groups were performed using the unpaired Student’s t test. When the comparison between groups required an adjustment by covariates, a univariate analysis of variance using a general linear factorial model was constructed. The χ^2^ test was used to compare categorical variables between groups.

Associations between continuous variables were described by the Pearson’s correlation coefficient. Multiple linear regression analysis was performed to identify the variables influencing cardiovascular risk (dependent variable). The independent variables used were those potentially related to cardiovascular risk, such as age, sex, smoking status, sedentarism and related medication (hypolipidaemic, antidiabetic and antihypertensive drugs).

In order to identify ucOC as an independent predictor of T2D, a multiple logistic regression model was performed. The usefulness of serum ucOC levels as a marker of T2D risk was analysed using a ROC curve. The AUC indicates the probability to predict an event. AUC values above 0.75 indicate a good predictive performance^59^.

Statistical significance was set at *p* < 0.05 (two tailed) and *p* < 0.10 for multiple linear regression analysis.
